# Fabrication and Dynamic Modeling of Bidirectional Bending Soft Actuator Integrated with Optical Waveguide Curvature Sensor

**DOI:** 10.1089/soro.2018.0061

**Published:** 2019-08-02

**Authors:** Wenbin Chen, Caihua Xiong, Chenlong Liu, Peimin Li, Yonghua Chen

**Affiliations:** ^1^ Institute of Robotics Research, State Key Laboratory of Digital Manufacturing Equipment and Technology, School of Mechanical Science and Engineering, Huazhong University of Science and Technology, Wuhan, China.; ^2^ Department of Mechanical Engineering, The University of Hong Kong, Hong Kong, China.

**Keywords:** soft actuator, bidirectional bending deformation, optical waveguide, trajectory tracking, sliding mode control, proprioceptive

## Abstract

Soft robots exhibit many exciting properties due to their softness and body compliance. However, to interact with the environment safely and to perform a task effectively, a soft robot faces a series of challenges such as dexterous motion, proprioceptive sensing, and robust control of its deformable bodies. To address these issues, this article presents a method for fabrication and dynamic modeling of a novel bidirectional bending soft pneumatic actuator that embeds a curvature proprioceptive sensor. The bidirectional bending deformation was generated by two similar chambers with a sinusoidal shape for reducing the internal dampness during bending deformation. An optical waveguide made from flexible poly (methyl methacrylate) material that is immune to the inlet pressure was embedded into the actuator body to measure its bending angle. A dynamic modeling framework based on step response and parameter fitting was proposed to establish a simple differential equation that can describe the nonlinear behavior of the soft actuator. Hence, a sliding mode controller is designed based on this differential equation and the Taylor expansion. The proposed dynamical model and the sliding mode controller were validated by trajectory tracking experiments. The performance of the bidirectional bending soft actuator, such as the linear output of the curvature sensor in different inflating patterns, the proprioceptive sensitiveness to the external environment, the output force, and large bending range under relatively small pressure, was evaluated by relevant experimental paradigms. Prototypes from the novel design and fabrication process demonstrated the soft actuator's potential applications in industrial grasping and hand rehabilitation.

## Introduction

The compliant nature endows the pneumatically actuated soft robots with significant advantages over the traditional rigid body robots in flexibility-required applications such as service robots that can safely interact with humans, rescue robots that conduct tasks in an unstructured environment, and medical robots for surgery, rehabilitative, or prosthetic purpose.^1–6^ The most studied soft robots are the soft actuators that have been designed to perform translation or rotation by axial or radial deformation.^5,7–9^ In many applications of soft actuator, the actuator is expected to have compact size and low hardness, capable of bidirectional bending with large bending angle and appropriate stiffness for object holding and manipulation. Furthermore, if a soft actuator can sense its deformation, it can then provide fundamental information for closed-loop feedback control. Typical tasks involving bidirectional bending, which have been reported in the literature, include autonomous slithering on the ground,^10,11^ the escape maneuvers in water,^12^ and the complex motion of human thumb.^8^

It has been shown previously that soft-bending actuators are constructed from polymeric^13^ or a combination of elastomeric (hyperelastic silicones) and inextensible materials (fabrics and fibers),^7,8^ and activated by pressurizing fluid media (liquid or gas). There are various designs of soft-bending actuators by varying the number of chambers and the cross-section shape. When considering a single chamber, the cross-section shape can be circular,^10,14^ rectangular,^15^ or semicircular^5,9,16^ where uniform bending is produced by asymmetrically constraining the extension of different layers of the air chamber.^17^ Nonuniform bending can be created by regulating the fabric distribution and winding of fibers along the actuators.^8^ More complex motions can be implemented by using multichambers.^4,16,18–21^

To implement bidirectional bending, some methods have been proposed by fabricating two^22,23^ or three^4^ individual actuators together, or casting several chambers together.^11,24–26^ The fluid pressurizes the partial actuators of the group enforcing them to bend toward the rest. Similarly, if the fluid only pressurizes the rest of the chambers, the group will bend toward another direction. Some research studies proposed more compact designs by integrating two similarly designed chambers into one actuator to replicate the symmetric agonistic and antagonistic motion under pressure without fiber or shell reinforcement.^12,27^ Considering the possible rupture or perforation in their walls under such design, a soft pneumatic actuator enveloped by a Yoshimura patterned origami shell that acts as an additional protection layer while providing specific bending resilience throughout the actuator's range of motion was reported.^28^ In another study, a negative air pressure was used to implement opposite bending corresponding to the deformation under positive air pressure.^17^

Regarding pose sensing of soft actuators, some research work employs the side-polished optical waveguide in which the intensity of transmitted light is related to flexion angle to design the proprioceptive curvature sensing element. The fabrication strategies include the use of macrobend stretch optical fibers^29^ and stretchable optical fibers^30–32^ for responding to multiple modes of deformation, the thin-film-designed waveguide applied in robot hands,^33^ engraved plastic optical fiber for bend measurement,^34–36^ and joint angle detection.^37–40^ The stiff magnetic sensors measure curvature of a bending segment by mapping the position of the magnet with respect to the stiff Hall element.^11,25^ Some other researchers integrate resistive sensors that basically measure a change in the current flow through conductive materials, such as the conductive ink,^41,42^ conductive polymers,^43^ eutectic galliumindium,^44^ and hydrogels.^45^ The capacitive sensing elements are also built to reveal a soft body bending angle by comparing the two sensory responses around the concave and the convex side of the bent body,^46^ or by detecting elongational strains.^47^ Most of the developed proprioceptive sensors detecting bending curvature, for example, the optical waveguide sensors made from elastomer material, are usually placed on the surface of soft object. They can only work well when none of the radial deformations are overlapped on the axial deformation because radial deformations will possibly distort the linear relationship between output voltage and the axial deformation corresponding to the bending angle.

Several issues still exist for the fabrication of bidirectional bending soft actuators integrated with sensing ability. First, the bidirectional soft actuator that works in constrained environment often requires a compact size to meet the task conditions such as the hand rehabilitative glove for stroke patients. Consequently, the external reinforced technique for protecting the actuator from perforation, such as the external shell reinforcement, will not be suitable for use in such constrained environments. Second, ordinary three-dimensional (3D) printing techniques have more difficulties in integrating the flexible sensor or interaction force sensor into the soft structure compared with the multistep molding techniques. Furthermore, the available materials for 3D printing are generally with greater hardness than that of the popular soft elastomers, hence it will consume more fluid pressure to overcome the inner stress of the material deformation. Third, most of the developed sensing elements integrated in the soft actuator for curvature detection are susceptible to the inflated pressure. Therefore, the calibrated relationship between the output voltage and curvature tends to be affected if multichambers are simultaneously inflated. This significantly affects performance in practical applications where closed-loop feedback control is required.

In this article, we present a new compact design of a soft pneumatic actuator with curvature proprioceptive ability. The actuator consists of two similar chambers with sinusoidal section shape, and each chamber is fiber reinforced by only winding around the wave peaks. The soft actuator is able to bend bidirectionally with large angle and exerts large tip force under a relatively small pressure. The optimized geometrical parameter is explored through finite element analysis (FEA) simulation. The customized optical waveguide with an intendedly roughened surface, made from poly (methyl methacrylate) (PMMA) material, is integrated into soft actuator as the proprioceptive curvature sensor. The optical waveguide is free of radial deformation and can provide steady linear output under air pressure. Because of the nonlinear behavior of the soft actuator, the modeling of dynamics and precise control of actuator's behavior are two fundamental issues of soft robotics. Therefore, a simplified dynamic modeling framework based on step response experiment is proposed. Considering that the dynamic model includes pressure-dependent parameters, the variable structure control strategy is studied and a competent sliding mode controller is developed. The proposed system was validated by a trajectory tracking experiment.

The main contributions of this article are as follows: (1) Differing from the popular reinforced fiber uniformly across the whole chamber, which results in relatively high material stress in pressure-induced bending deformation, the wave chamber with sinusoidal section shape proposed in this research is fiber reinforced only around the wave peak. This design enhances the bidirectional bending ability of the soft actuator in lower air pressure. (2) Through step response experiment, the dynamic modeling framework considering the pressure-dependent parameters is developed toward reproducing the nonlinear behavior of the soft actuator. The technique of Taylor expansion is used to locally linearize the control variable and further establish the model-based variable structure controller. By embedding the plastic optical fiber curvature sensor, the feedback control is implemented.

This article is organized as follows: the objective of this work is given in the Objectives section. In the Materials and Methods section, the fabrication of the soft actuator and the integration of optical waveguide are first provided. Then, the influence and mechanism of the section parameter of the chamber on bending deformability are studied. Third, the curvature description and the measuring principle by using the optical waveguide are presented. Finally, the dynamic modeling strategy of the soft actuator is discussed, and the variable structure sliding mode controller is designed for closed-loop feedback control. In the Results section, we verify the performance of the soft actuator in bidirectional bending, sensor sensitivity, output force, and angular trajectory control by different experimental paradigms.

## Objectives

In this work, we address two main issues: (1) develop a soft actuator with bidirectional bending ability and proprioceptive curvature sensing function that is robust in large air pressure; (2) formulate a general framework for dynamic modeling of the soft actuator and implement precise curvature control with only proprioceptive information. Around these two objectives, the proposed design strategy will be validated through experiment. The related geometrical parameters of the chamber for desired bending deformation will be investigated. The performance of the optical waveguide based on PMMA material in measuring the general curvature, such as the robustness and output linearity under free deformation, will be studied. The dynamic model and the feedback control through the proprioceptive curvature will be developed.

## Materials and Methods

### Fabrication of the soft actuator

**FIG. 1.**
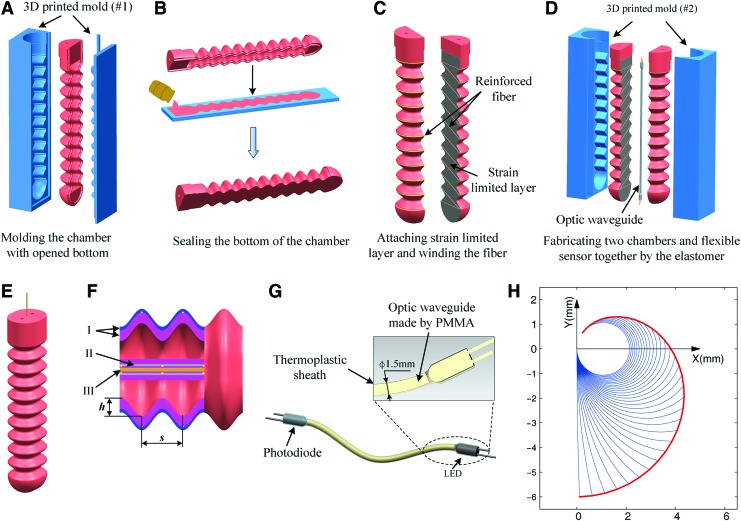
Typical fabrication steps of the soft actuator, structure of the optical waveguide and freely continue bending deformation of the soft actuator. **(A)** Fabricating the open chamber with a sinusoidal shape. **(B)** Closing the open side of the chamber with an elastomer sheet. **(C)** Attaching the strain limited layer and winding the reinforced fiber. **(D)** Encapsulating the two fiber-reinforced chambers and flexible sensor together. **(E)** The final soft actuator with bidirectional bending ability. **(F)** The inner structure of the soft actuator: (I) the wall of the chamber corresponding to different fabrication steps, (II) the strain limited layer (woven fiberglass), and (III) the optical waveguide. The two main geometrical parameters for the chamber with the sinusoidal wave: pitches *s* and amplitude *h*. **(G)** The customized optical waveguide with transmit probe (Tx) and receive detector (Rx). **(H)** Bending deformation of the neural axis (optical waveguide) of the soft actuator. Color images are available online.

The structure and main component of the soft-bending actuator are shown in Figure 1. The elastomeric body was fabricated by the silicone composite (ELASTOSIL^®^ RT 622 A). The soft body comprises two identical chambers, which were cast together through the middle layer that was integrated with nonstretchable fiberglass fabric. The shape of the chamber was a sinusoidal wave with multiperiod, which was radially reinforced by the Kevlar fibers incorporated into the elastomeric material (Fig. 1C). The fibers were only wound around the peak, and then crossed through the bottom in the normal and inverse direction. Such fabrication strategy constrains the radial bulging around the wave peak but let groove region between peaks freely bulge, which significantly improves the natural axial elongation ability of the elastomeric body. Furthermore, the fabrication decreases the inner squeezing stress from material stacking during compression. When inflating one of the two chambers, the elongation of stretching side chamber and the shortening of the compressed side chamber are then transformed to the lateral bending deformation under the constraint of middle strain limited layer.

### The integration of curvature proprioceptive function

To measure the general curvature of the soft actuator under inflated pressure, the optical fiber made from transparent PMMA material was fabricated to be intentionally lost. As light propagates through it, some laterally refract the environment, and some repeatedly reflect the end port. The more it is deformed, the more the light is lost. Thus the bending deformation can be indicated by measuring the light power loss. The relationship between curvature and power loss can be calibrated by individual experiment. Considering bendable but not stretchable feature of the PMMA, the optical waveguide was embedded into the middle layer whose length keeps constant due to the limitation of the glass fiber (Fig. 1D). It must be noted that the inflated chambers will provide pressure force to the optical waveguide, which will introduce unexpected radial deformation of the waveguide if it is fabricated by the transparent elastomeric silicone.^30^ Such radial deformation will bring additional light loss, and further affects the original relationship between curvature and light loss. Hence the PMMA-based optical waveguide was chosen as the curvature sensor, for its incompressible feature under air pressure.

To fabricate the sensory waveguide, the thermoplastic sheath was used as the jacket to steadily hold the probes relative to the ends of waveguide (Fig. 1G). As the highly absorptive composite, the thermoplastic sheath insulates the optical waveguide from the nonuniform local deformation introduced by the tensioned Kevlar fibers in inflation.

### Curvature-based configuration description

For the traditional rigid link-based manipulator, the configuration of the mechanism is determined by the joint angle or prismatic displacement. For the soft actuator, the curvature is the independent variable. The chosen variable is appropriate because the input pressure to the soft segment has a direct effect on the curvature. In this work, the curvature is measured on the neural axis of the soft actuator whose length approximately keeps constant and is equal to the initial length of the actuator. The free bending of the neural axis is a uniform arc whose curvature can be measured by the integrated sensor. Thus, the free continual bending deformation of the soft actuator can be seen as the time-varying arc whose curvature continually varies from the great value to the small value. If the level length and the curvature of the neural axis of the soft actuator are denoted by *l*_0_ and $k ( t )$, respectively, then the subtended angle $\theta ( t )$ at the instantaneous center of the circle corresponding to the arc is

\begin{align*}
\theta ( t ) = {l_0}k ( t ). \tag{1}
\end{align*}

Because the curvature is proportional to the subtended angle, it is reasonable to model the configuration of the soft actuator by the subtended angle instead of the curvature. Thus the generalized coordinate to describe the system configuration is the subtended angle. As an example of this point, the continual deformation of neural axis of a soft actuator with constant length 6 mm, that is, continual variation of curvature *k*, is shown in Figure 1H by varying the subtended angle $\theta$ from 0° to 330°.

### Section parameter of chamber on bending deformability

As for section shape with the sinusoidal wave, there are several shape parameters that determine the dampness and affect the bending ability for the given inlet pressure, such as the lateral wall thickness, middle layer thickness, pitch, and the amplitude of the sinusoidal wave. For real application, it is desired that the actuator can bend bidirectionally under small pressure while produces acceptable output force. To reduce the analysis complexity and find the general influence, we mainly consider the following two significant parameters (Fig. 1F): pitch *s* and the amplitude *h*. The FEA simulation was used to study such two parameters on the bending performance.

**FIG. 2.**
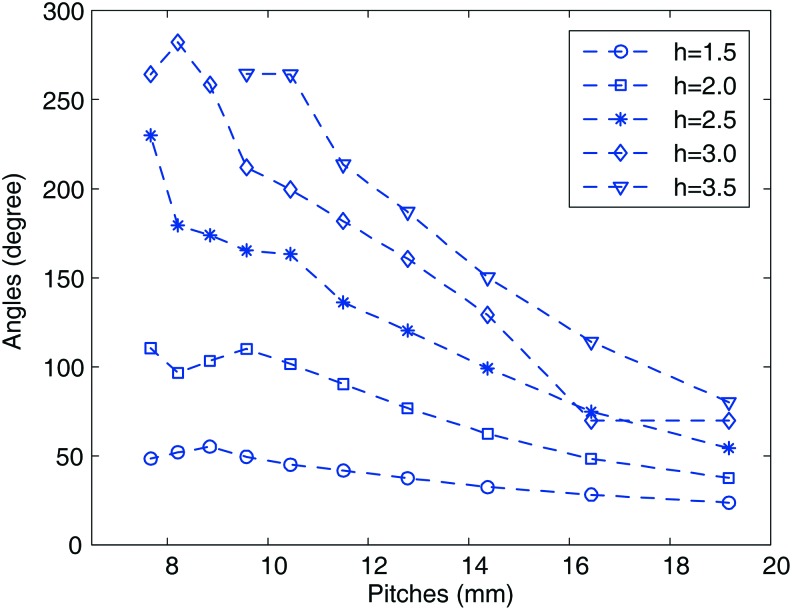
The subtended angle of the soft actuator for different pitches under the specified amplitude of the sinusoidal wave. Color images are available online.

The thickness and longitudinal length of the chamber were fixed for all simulations; that is, 3 and 115 mm. For the specified pitch and amplitude of the wave, the soft actuator was pressurized by 0.1 MPa, and the final subtended angle was recorded. This simulation was repeated under different pitches and amplitudes. In the simulation experiment, the amplitude varied from 1.5 to 3.5 mm at step 0.5 mm. For each amplitude, the pitch was varied to let the number of waves of the chamber vary from 6 to 15. The simulation result is given in Figure 2.

**FIG. 3.**
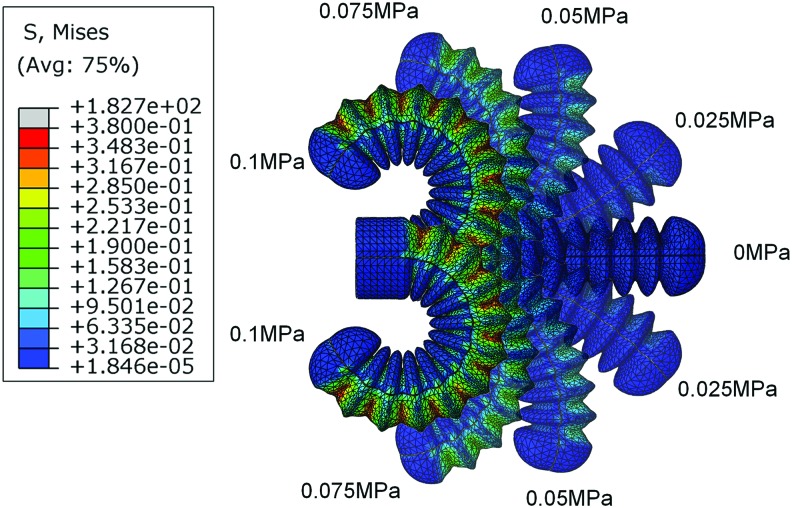
The simulated bidirectional bending behavior under different pressure when separately inflating the two chambers. Color images are available online.

For the same air pressure, the greater subtended angle represents the relatively low inner dampness and higher bending ability. As shown in Figure 2, the pitch of the sinusoidal wave is approximately monotonically decreasing with the subtended angle. Basically, the smaller pitch of the sinusoidal wave brings greater bending deformation under the same pressure. Meanwhile, for the specified pitch, the greater amplitude generally brings better bending ability. The soft actuators with greater amplitude are basically easy to bend compared with the soft actuator with smaller amplitudes. In the specified range of the parameters, when one chamber was inflated with 0.1 MPa pressure, the greatest subtended angle reached 280° under pitch 8.5 mm and amplitude 3.5 mm, the least subtended angle was just 50° under pitch 8.5 mm and amplitude 1.5 mm. For great pitches, the variation of amplitudes shows a relatively small influence on the bending ability. However, the bending ability is more sensitive to the amplitude when fabricating the soft actuator with a small pitch. The illustrative steady bending deformation of the soft actuator with pitch 8.5 mm and amplitude 3.5 mm is shown in Figure 3 when individually inflating the two chambers.

Although the geometrical parameters are discretized in a very limited range, the general tendency of the bending under the different geometrical parameters is effectively revealed. The greater amplitude and smaller pitch endow the soft actuator better bending ability.

### Metric of the curvature by the optical waveguide

If the output power of the waveguide under no deformation is defined as the baseline power *V*_0_, the power loss of the waveguide is defined as the following logarithm function^30^:

\begin{align*}
I = 10{ \log _{10}} ( {{{V_0}} \mathord{ \left/ { \vphantom {{{V_0}} V}} \right. \kern- \nulldelimiterspace} V} ) , \tag{2}
\end{align*}

where *V* is the output power. From this definition, the output power loss *I* is zero under no deformation, $I > 0$ with increasing deformation. In this work, the voltage of the photodiode was used to indicate the output power.

**FIG. 4.**
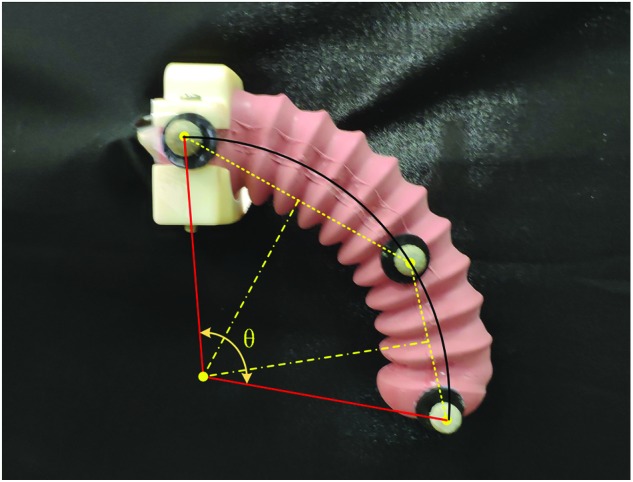
The principle to calculate the subtended angle for sensor calibration. Color images are available online.

**FIG. 5.**
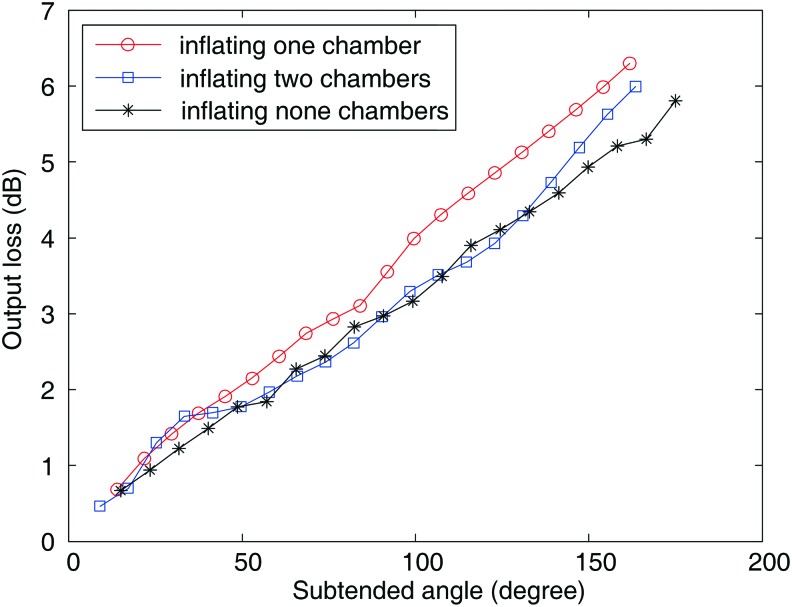
The linear relationship between the subtended angle and the power loss. Color images are available online.

The linearity of the waveguide under different inflation patterns is essential for the usability as the curvature sensor. To specify such property of the waveguides, three inflation patterns are studied: (I) inflating only one chamber, (II) inflating both the chambers, and (III) bending without inflation. For the bending under inflation (patterns I and II), the camera was used to capture the steady image, which was then used to measure the subtended angle. Three markers prestuck on the neural axis, that is, one at the middle and two at the end, were used to find the arc center, which was further used to form the subtended angle. The measuring principle is shown in Figure 4. For the bending without inflation (pattern III), the 3D printed molds with a curved groove to contain the soft actuator were used to shape it to the desired angle. The voltage from the photodiode was simultaneously recorded. The corresponding relationship between the subtended angle and the output power loss is shown in Figure 5.

The linear relationship between the subtended angle of the soft actuator and the output power loss is highly significant in the cases of inflating only one individual chamber or both the chambers. The radial incompressibility of the PMMA material eliminates the complex local deformation introduced by the inflated air pressure from the optical waveguide and helps maintain the linear relationship. This characteristic assigns the PMMA waveguide with superiority over the flexible sensor based on conductive ink and the optical waveguide made of elastomeric silicone.

Benefiting from the high linearity between subtended angle and the output power loss, the power loss can be utilized as an indication of the bending angle of the soft actuator through calibration using the experimental data shown in Figure 5.

### Dynamics response modeling

**FIG. 6.**
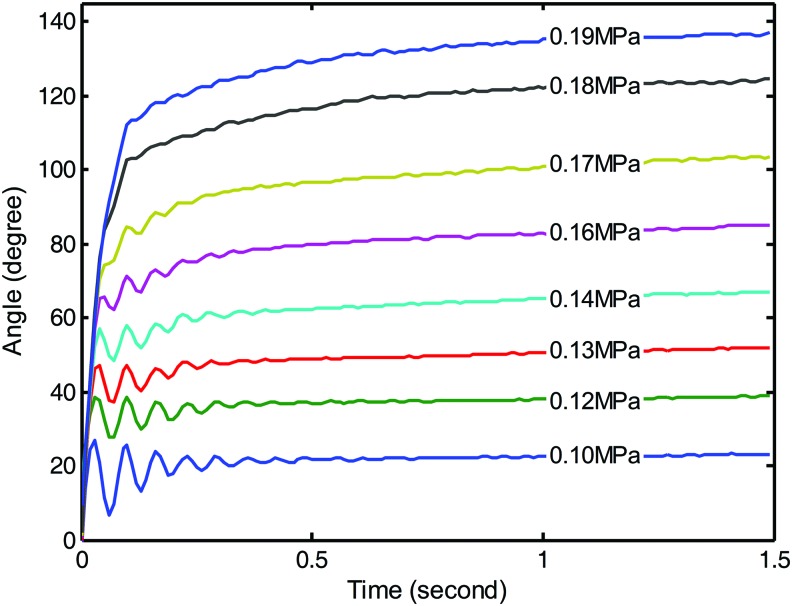
The sampled dynamic step response under different pressure. Color images are available online.

The exact model of the soft actuator is a challenging issue due to the high nonlinearity of the interaction between the elastomer and pressured fluid. However, we can explore the fundamental principle of the system by observing the step response under different pressure. Because of the completely symmetrical structure of the soft actuator, we only consider the dynamic response in the case of inflating one chamber. The illustrative dynamic responses under different pressure are given in Figure 6.

According to the profile of the step response curve, the system shows both features of decaying oscillation and the exponential decaying toward the steady angle. The oscillation component could be obtained from the internal resonance of the soft actuator. It is reasonable to infer that the soft actuator is similar to a compositive system. The compositive system is formed by superimposing two individual systems that have complex conjugate pole pair and a real pole, respectively, on the left-half complex plane.^48^ The complex conjugate pole pair on the left-half complex plane corresponds to the decaying oscillation feature of an underdamping system, whereas the real pole corresponds to the exponential decaying feature of an overdamping system. Thus, the general response of the soft actuator under step inflation can be viewed as a third-order system (three poles on the left-half complex plane).

When modeling the system, keeping the oscillation term is helpful to improve the high-frequency motion ability. However, the oscillation term will bring more parameters and increase the model complexity. Furthermore, the estimation of modeling uncertainty will be difficult and further increase the complexity of the controller. To reduce the modeling complexity while keeping the system basic feature, we keep the real part of the complex conjugate pole pair. Then, the system is approximated by superimposing two overdamping systems. The introduced error in this approximation is the model uncertainty, which can be included in the variable structure controller.^49^ Under this framework, the response of the system can be fitted by the following time domain equation:

\begin{align*}
\theta = {C_1}{e^{ - { \lambda _1}t}} + {C_2}{e^{ - { \lambda _2}t}} + {C_3} , \tag{3}
\end{align*}

where ${C_1} \in {{ \rm{R}}^ - }$, ${C_2} \in {{ \rm{R}}^ - }$, and ${C_3} \in {{ \rm{R}}^ + }$ are dependent on the initial conditions and the inlet pressure. Especially, *C*_3_ is the steady-state angle. ${ \lambda _1}$ and ${ \lambda _2}$ are both positive and also depend on the pressure. As different step input pressure brings a different response curve, this formula is a general expression. For the clusters of response curves, the least-square fitting technology can be used to establish dependent functions about coefficients ${ \lambda _1}$, ${ \lambda _2}$, and *C*_3_ to the pressure *u*.

The step response given in Equation (3) is actually the solution of a second-order differential system [Eq. (4)] in the time domain. Although Equation (4) is similar to the work in the literature^10,22,50^ from appearance, it is with a different meaning. The system given in Equation (4) is a nonlinear time-invariant system due to the pressure-dependent coefficients:

\begin{align*}
\ddot \theta + \left( {{ \lambda _1} + { \lambda _2}} \right) \dot \theta + { \lambda _1}{ \lambda _2} \theta = { \lambda _1}{ \lambda _2}{C_3} , \tag{4}
\end{align*}

where *u* is the outlet pressure from the pneumatic proportional valve into the chamber. It must be noted that the formula (4) is just roughly approximation, since there is fitting error when determining the relationship between coefficients ${ \lambda _1}$, ${ \lambda _2}$, *C*_3_ and the pressure *u*. However, it is possible to estimate the boundary of such model uncertainty and implement precise angle control by using the variable structure control strategy.

If the $\Delta { \lambda _1}$ and $\Delta { \lambda _2}$ are defined as the variation of ${ \lambda _1}$ and ${ \lambda _2}$, substitute ${ \lambda _1}$ with ${ \lambda _1} + \Delta { \lambda _1}$, ${ \lambda _2}$ with ${ \lambda _2} + \Delta { \lambda _2}$, we can obtain the following formula $\Delta r \;$ to represent the model uncertainty:

\begin{align*}
\begin{split} \Delta r = &- \left( { \Delta { \lambda _1} +
\Delta { \lambda _2}} \right) \dot \theta + \\ &\ \left( { \Delta
{ \lambda _1} \Delta { \lambda _2} + { \lambda _1} \Delta {
\lambda _2} + { \lambda _2} \Delta { \lambda _1}} \right) \cdot
\left( {{C_3} - \theta } \right)
\end{split}
. \tag{5}
\end{align*}

When providing system performance requirement, such as the maximum speed and range of input pressure, the $\Delta r$ can be generally estimated.

Considering the compromise between the model simplicity and fitting accuracy, the three parameters ${ \lambda _1}$, ${ \lambda _2}$, and *C*_3_ can be fitted as the function of outlet pressure *u* from the pneumatic proportional valve:

\begin{align*}
\begin{split} & { \lambda _1} = {a_1}u + {a_0} \\ & { \lambda _2} = {b_1}u + {b_0} \\ & {C_3} = {c_1}{u^2} + {c_0}u , \\\end{split}
\tag{6}
\end{align*}

where ${a_0} , {a_1} , {b_0} , {b_1} , {c_0} , {c_1}$ are constant coefficients depending on the data of the step response experiment.

After substituting Equation (6) into Equation (4), we obtain the following formulas:

\begin{align*}
\ddot \theta = {f_1} \left( u \right) \dot \theta + {f_2} \left( u \right) \theta + {f_3} \left( u \right) , \tag{7}
\end{align*}

where

\begin{align*} 
& {f_1} ( u ) = - ( {a_1} + {b_1} ) u - {a_0} - {b_0} \\ & {f_2} ( u ) = - {a_1}{b_1}{u^2} - ( {a_1}{b_0} + {a_0}{b_1} ) u - {a_0}{b_0} \\ & {f_3} ( u ) = {a_1}{b_1}{c_1}{u^4} + ( {a_1}{b_0}{c_1} + {a_0}{b_1}{c_1} + {a_1}{b_1}{c_0} ) {u^3} \\ &\quad \quad \quad { \rm{ }} + ( {a_1}{b_0}{c_0} + {a_0}{b_1}{c_0} + {a_0}{b_0}{c_1} ) {u^2} + {a_0}{b_0}{c_0}u. \\
\end{align*}

The system [Eq. (7)] is related to the high order of the system control and is not common for designing the sliding mode controller. Considering that the input voltage of the valve was always varying in very limited range, it is reasonable to reduce the order of the system control by using the Taylor expansion. Here we use the first-order Taylor expansion to approximate the high-order term ${f_2} ( u )$ and ${f_3} ( u )$, and then Equation (7) can be approximated to the following expression:

\begin{align*}
\begin{split}& \ddot \theta = \left( {{f_1} \left( {{u_0}} \right) + f\prime _1 \left( {{u_0}} \right) \cdot \left( {u - {u_0}} \right) } \right) \dot \theta + \\ & \, \, \, \, \, \, \, \, \; \; \; \left( {{f_2} \left( {{u_0}} \right) + f \prime _2 \left( {{u_0}} \right) \cdot \left( {u - {u_0}} \right) } \right) \theta + \\ & \; \; \; \; \; \; \;{f_3} \left( {{u_0}} \right) + f \prime _3 \left( {{u_0}} \right) \cdot \left( {u - {u_0}} \right) ,\end{split}
 \tag{8}
\end{align*}

where *u*_0_ is the reference input pressure and

\begin{align*}
\begin{split} & {{f \prime } _1} ( u ) = - ( {a_1} + {b_1} ) \\ & {{f \prime } _2} ( u ) = - 2{a_1}{b_1}u - ( {a_1}{b_0} + {a_0}{b_1} ) \\ & {{f \prime } _3} ( u ) = 4{a_1}{b_1}{c_1}{u^3} + 3 ( {a_1}{b_0}{c_1} + {a_0}{b_1}{c_1} + {a_1}{b_1}{c_0} ) {u^2} \\ &\quad\quad\quad { \rm{ }} + 2 ( {a_1}{b_0}{c_0} + {a_0}{b_1}{c_0} + {a_0}{b_0}{c_1} ) u + {a_0}{b_0}{c_0}. \\\end{split}
\tag{9}
\end{align*}

Thus, further reformulating Equation (8) to the normal form:

\begin{align*}
\ddot \theta = g \left( { \dot \theta , \theta } \right) + h \left( { \dot \theta , \theta } \right) \cdot \left( {u - {u_0}} \right) , \tag{10}
\end{align*}

where

\begin{align*}
\begin{matrix}{g \left( { \dot \theta , \theta } \right) = {f_1} \left( {{u_0}} \right) \dot \theta + {f_2} \left( {{u_0}} \right) \theta + {f_3} \left( {{u_0}} \right) } \\ {h \left( { \dot \theta , \theta } \right) = f \prime _1 \left( {{u_0}} \right) \dot \theta + f\prime _2 \left( {{u_0}} \right) \theta + f\prime _3 \left( {{u_0}} \right) } \\ \end{matrix}. \tag{11}
\end{align*}

### Sliding mode controller design

The dynamic model of the soft actuator carries pressure-dependent parameter whose variation and error bring the nonlinear feature for the system. As a systematic approach to the problem of maintaining stability and consistent performance in the face of modeling uncertainty, the variable structure sliding mode control strategy is especially suitable to design the controller for the system of the soft actuator.

Let ${x_1} = \theta$, ${x_2} = \dot \theta$, and considering the system error, the differential equation of the system model [Eq. (10)] is written as

\begin{align*}
\begin{split}&{{{ \dot x}_1} = {x_2}} \\ &{{ \dot x}_2} = g
\left( {{x_1} , {x_2}} \right) + h \left( {{x_1} , {x_2}} \right)
\cdot \tilde u + \Delta r
\end{split}
, \tag{12}
\end{align*}

where the ${ \tilde u}= u - {u_0}$ is assigned as the system control and is limited to a small range in the controller, so that small variation around the reference pressure *u*_0_ will occur for using the Taylor expansion. $\Delta r$ is a manually added term to consider the system modeling error, and its detailed expression is given in Equation (5).

The tracking problem for the system is to find a control law ${ \tilde u}$, such that the closed-loop system satisfies ${ \lim \nolimits_{t \to \infty }}{x_d} ( t ) - x ( t ) = 0$, where ${x_d} ( t )$ is the target trajectory. First, let

\begin{align*}
\begin{split} & e = {x_d} - {x_1} \\ & s = ce + e
\end{split}
, \tag{13}
\end{align*}

where *x_d_* is the desired trajectory, *c* is the positive constant, and is used to adjust for the weight difference between the bending angle and bending velocity. The Lyapunov function can be defined as $V \;{ \rm{ = }} \;{{{s^2}} \mathord{ \left/ { \vphantom {{{s^2}} 2}} \right. \kern- \nulldelimiterspace} 2}$. And then, the derivative of this function is

\begin{align*}
\dot V = s \left( {c \dot e + {{ \ddot x}_d} - g - h { \tilde u}- \Delta r} \right). \tag{14}
\end{align*}

Thus, to $\dot V < 0$, the sliding mode controller can be designed as

\begin{align*}
 { \tilde { u } } = \frac { 1 } { h } \left( { c \dot e + { { \ddot x } _d } - g + \eta s + D \cdot sgn \left( s \right) } \right) , \tag { 15 } 
\end{align*}

where $\eta$ is the positive constant which is used to control the convergence speed of the difference between practical trajectory and the desired trajectory. ${ \mathop{ \rm sgn}} ( \cdot )$ is the sign function, which is assigned to be +1 for the positive variable and −1 for the negative variable. *D* is the maximum fluctuation range of system model error $\Delta r$, and it is given by

\begin{align*}
\begin{matrix}{ \left\vert { \Delta r} \right\vert \le \left\vert { \left( { \Delta { \lambda _1} + \Delta { \lambda _2}} \right) \dot \theta } \right\vert + } \\ { \left\vert { \Delta { \lambda _1} \Delta { \lambda _2} + { \lambda _1} \Delta { \lambda _2} + { \lambda _2} \Delta { \lambda _1}} \right\vert \cdot \left\vert {{C_3} - \theta } \right\vert = D} \\ \end{matrix} \;. \tag{16}
\end{align*}

## Results

Considering the compromise of easy fabrication and performance, soft actuator with pitch 8.5 mm and amplitude 3.5 mm was fabricated to verify the proposed design and modeling framework. The electric proportional pressure valve with high rate of flow (VPPM-NPT, FESTO Ltd.) was used in the following tests.

### Sensor sensitivity test

To test the sensitivity of the optical waveguide under external disturbance, we conducted the lateral scanning experiment to detect the contour of the object.^30^ To steadily contact the surface for improving the sensitivity, one of the chambers was pressurized with 0.1 MPa to bend a probable angle and keep a constant stiffness. Three 3D printed objects with different surface contours were placed on a movable platform. The proximal end was placed on the fixed platform, and the distal end of the soft actuator kept contacting the surface. When the object moved ahead in average speed 0.2 cm/s, the subtended angle was recorded. The height profile of the contacted surface was reconstructed by the varying signal from the optical waveguide. The fourth-order low-pass Butterworth filter with 10 Hz cutoff frequency was used to proceed the noise from the sensor. Before the lateral scanning, we carried out a calibration procedure in which the soft actuator moved along an inclined plane. The obtained relationship between the height of the contact point and the angle of the soft actuator was used to reconstruct the contour in the lateral scanning experiment.

**FIG. 7.**
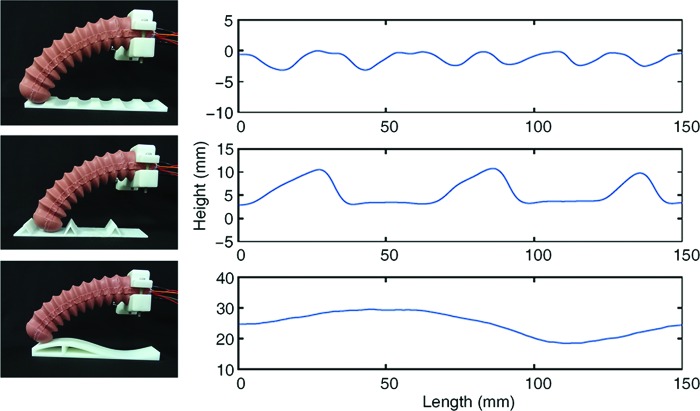
Contour detection of different surfaces: concave wave, triangle wave, and sinusoidal wave (*left*); the corresponding reconstructed contour (*right*). Color images are available online.

The experimental paradigms and the lateral scanning results are shown in Figure 7. From these data, we observe that the soft actuator can distinguish multishapes with the concave surface, triangular wave, and sinusoidal wave. Such experiment demonstrates the ability of the PMMA-based optical waveguide to dynamically sense time-varying disturbance acting on the soft actuator.

### Output force test

**FIG. 8.**
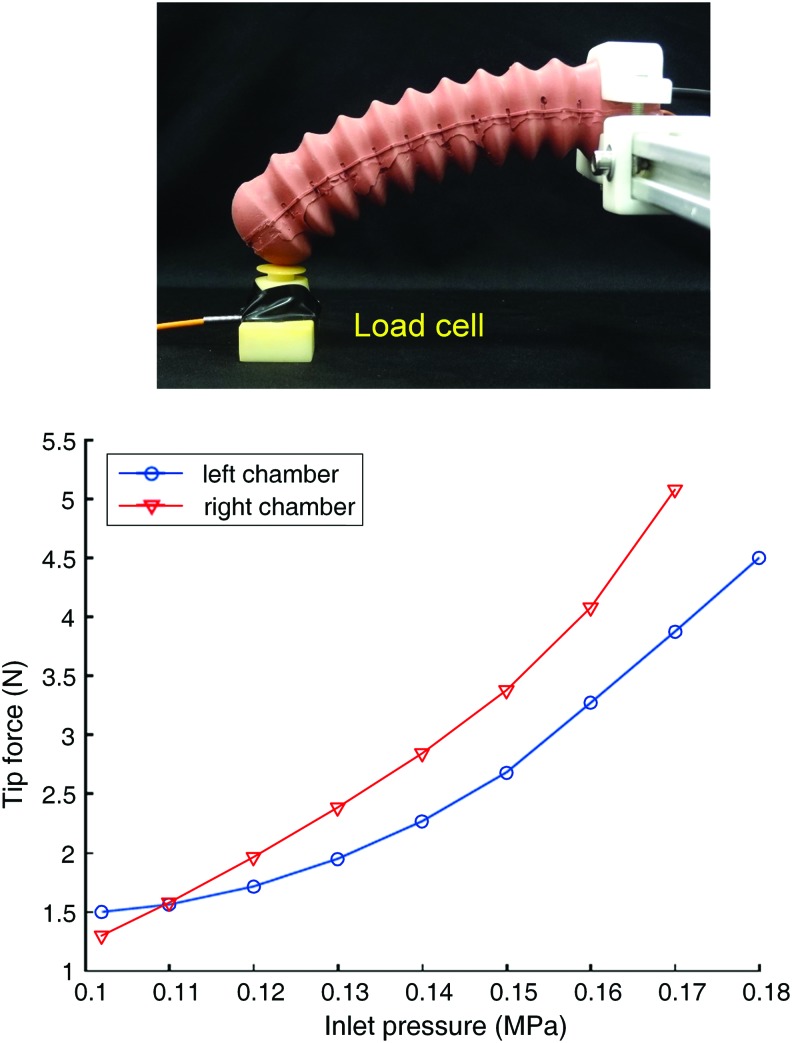
The output force test. The *upper image* shows an experimental setup and the *lower figure* represents the exerted tip force under increasing air pressure. Color images are available online.

To validate the output force of the soft actuator, we collected the tip force exerted by the actuator through a compression load cell (FC10-50N; Forsentek Co., Limited, China) (Fig. 8). The tip of the actuator was in contact with the load cell. The proximal end of the actuator was mounted on the platform and was linked to the air source. During pressurization, the actuator exerted force to the load cell through flexion, which was different from the blocked force obtained by constraining the curvature of the pressurized soft actuator.^27^ When one chamber was pressurized, the other one was kept in nonpressurized status. The pressure increased in steps of 10 kPa and the tip force was recorded after the pressure holding for 20 s in each step to obtain the steady status of the soft actuator. The experiment results are provided in Figure 8. The force at the inlet pressure 0.17 MPa is up to 5.5 N. Due to the minor difference in manual fabrication, the output force of the two pressurized chambers under the same pressure is not the same. However, the output force from the two pressurized chambers is ascending with the increased pressure in the range of 0.18 MPa.

### Controller-related parameter determination

Before validating the sliding mode controller of the system under the proprioceptive curvature sensor, there are two groups parameters need to be predetermined. One group involves the system dynamics-related parameters; that is, ${ \lambda _1}$, ${ \lambda _2}$, and *C*_3_. Another group involves the constant parameters related to the controller; that is, *D*, *c*, and $\eta$.

**FIG. 9.**
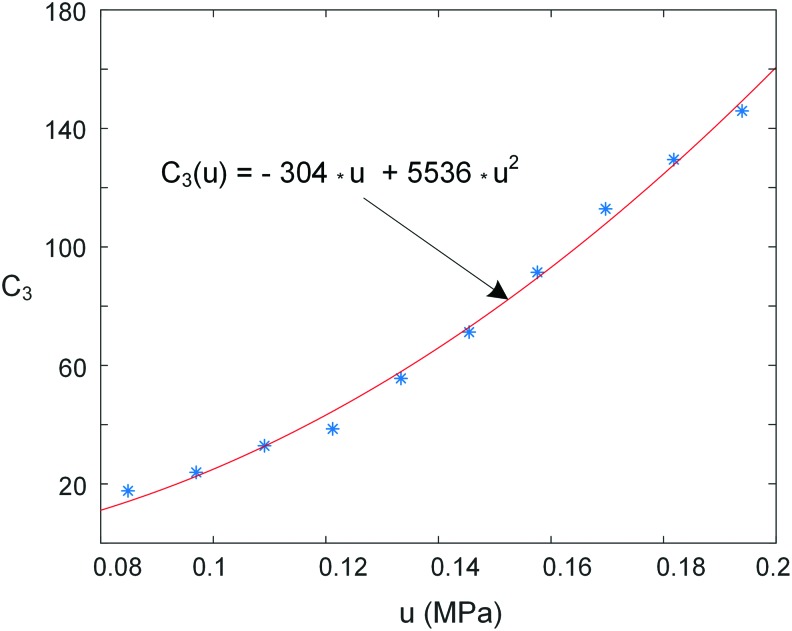
The relationship between the parameter *C*_3_ and input pressure *u*. Color images are available online.

**FIG. 10.**
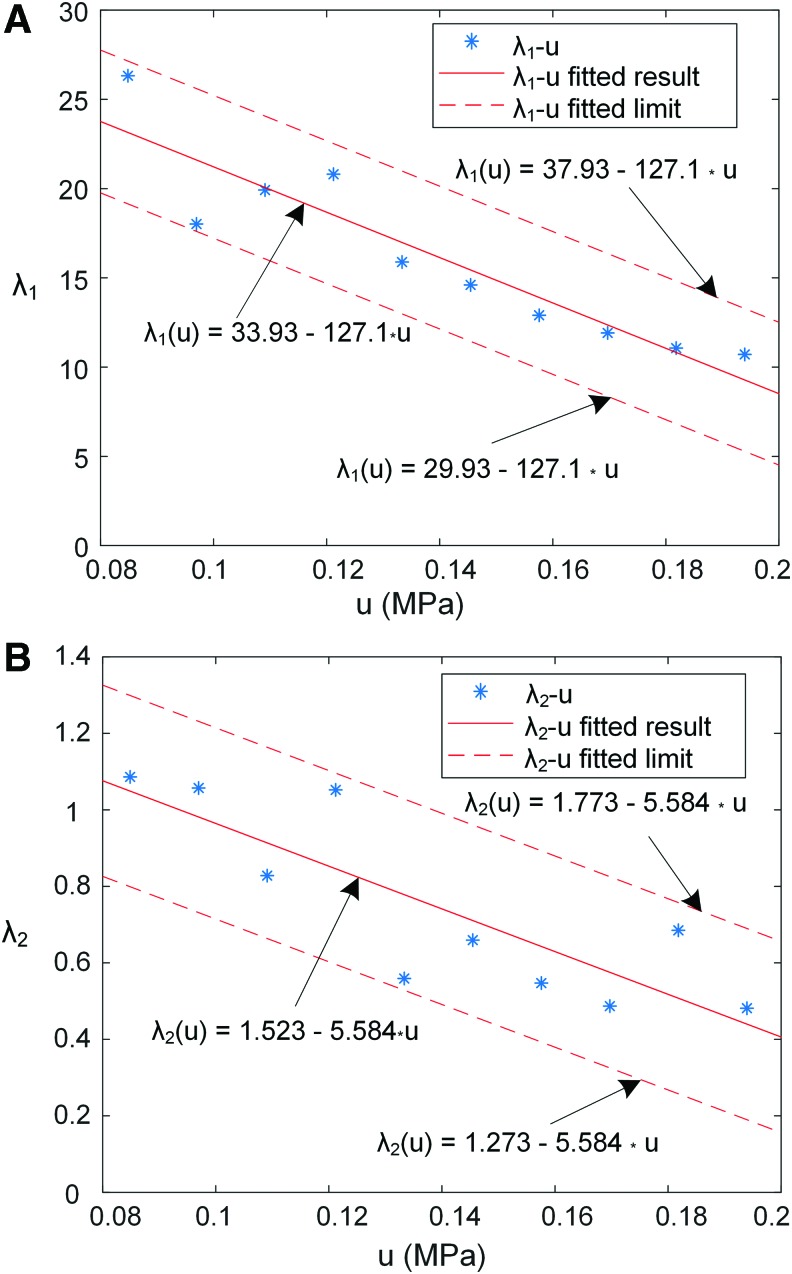
The relationship between parameters *λ*_1_, *λ*_2_, and input pressure *u*. **(A)** *λ*_1_ versus *u*, and **(B)** *λ*_2_ versus *u*. Color images are available online.

In this work, the maximum pressure of the chamber was limited to 0.2 MPa. The fitting procedure was performed to find the relationship between ${ \lambda _1}$, ${ \lambda _2}$, *C*_3_ and the input pressure *u* in Equation (6) to obtain the system dynamic equation. For a specified input pressure *u*, the least-squares technique was employed to obtain the particular value of the parameters${ \lambda _1}$, ${ \lambda _2}$, and *C*_3_. The fitting procedure was repeated for different step responses with input pressure from 0.08 to 0.2 MPa in steps of 0.01 MPa. Then, a collection of ${ \lambda _1}$, ${ \lambda _2}$, and *C*_3_ under different input pressure *u* was obtained (Figs. 9 and 10). Thus, the corresponding relationship between the three parameters and the inlet pressure can be approximately established.

Because *D* is the maximum fluctuation range of system model error $\Delta r$, its specific value depends on the requirement of maximum speed and range of input pressure. From the general performance of the system, it is supposed that $\left\vert { \dot \theta } \right\vert$ $\le {{400^ \circ } \mathord{ \left/ { \vphantom {{400^ \circ } s}} \right. \kern- \nulldelimiterspace} s}$, $\left\vert {{C_3} - \theta } \right\vert \le 180^ \circ$. As shown in Figure 10, $\left\vert { \Delta { \lambda _1}} \right\vert \le 4$, $\left\vert { \Delta { \lambda _2}} \right\vert \le 0.25$. In the range of 0.2 MPa pressure, the maximum nominal values of ${ \lambda _1}$ and ${ \lambda _2}$ are 34 and 1.5, respectively. Then, the *D* approximates to ${{{ \rm{4500}}^ \circ } \mathord{ \left/ { \vphantom {{{ \rm{4500}}^ \circ } {{{ \mathop{ \rm s} \nolimits} ^2}}}} \right. \kern- \nulldelimiterspace} {{{ \mathop{ \rm s} \nolimits} ^2}}}$.

For the *c* and $\eta$, these two coefficients were tuned by hand to maximize the performance in step response, focusing on minimizing rise time and limiting subsequent oscillations. In this work, we used $c = {{10} \mathord{ \left/ { \vphantom {{10} { \mathop{ \rm s} \nolimits} }} \right. \kern- \nulldelimiterspace} { \mathop{ \rm s} \nolimits} }$ and $\eta = {{50} \mathord{ \left/ { \vphantom {{50} { \mathop{ \rm s} \nolimits} }} \right. \kern- \nulldelimiterspace} { \mathop{ \rm s} \nolimits} }$.

### Model validation and controller performance test

To validate the proposed dynamic model and controller by using the proprioceptive angle sensor, the model step response and trajectory tracking experiment were further performed.

**FIG. 11.**
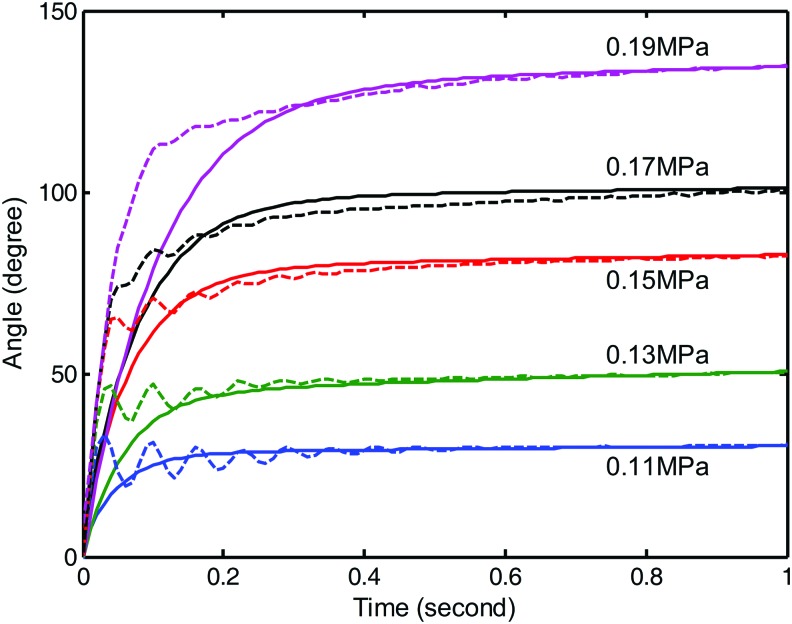
Comparison of model output and the natural response of soft actuator by step input at different pressure. The *solid lines* represent the model output, while the *dotted lines* indicate the natural response when inflating one chamber. For the same excitation pressure, results from the model and soft actuator are marked by the same color. Color images are available online.

First, the dynamic performance of the proposed model and controller for the soft actuator was verified by the step response of different pressure inputs. The output of the model and the experimental data from 0.11 to 0.19 MPa are compared and shown in Figure 11. The corresponding values of the parameters ${ \lambda _1}$, ${ \lambda _2}$, and *C*_3_ in the model were determined by the specified pressure through an averagely fitted formula shown in Figures 9 and 10. Through integration along the time, the model outputs were obtained. Due to the order reduction and parameters fitting error, the model shows the overdamping feature without any overshoot and oscillation. The model's rising speed to the level of steady angle is generally slower than the natural response of the soft actuator. However, the time taken to reach the steady angle of the model is always shorter than the time of natural response. It is inferred from the overdamping characteristic of the model that the system will show good performance in lower frequency and would be weak in higher frequency. Generally speaking, the model output can basically match the natural response of the soft actuator. The trajectory difference found in the experiment provides the tuning space for the sliding mode controller.

**FIG. 12.**
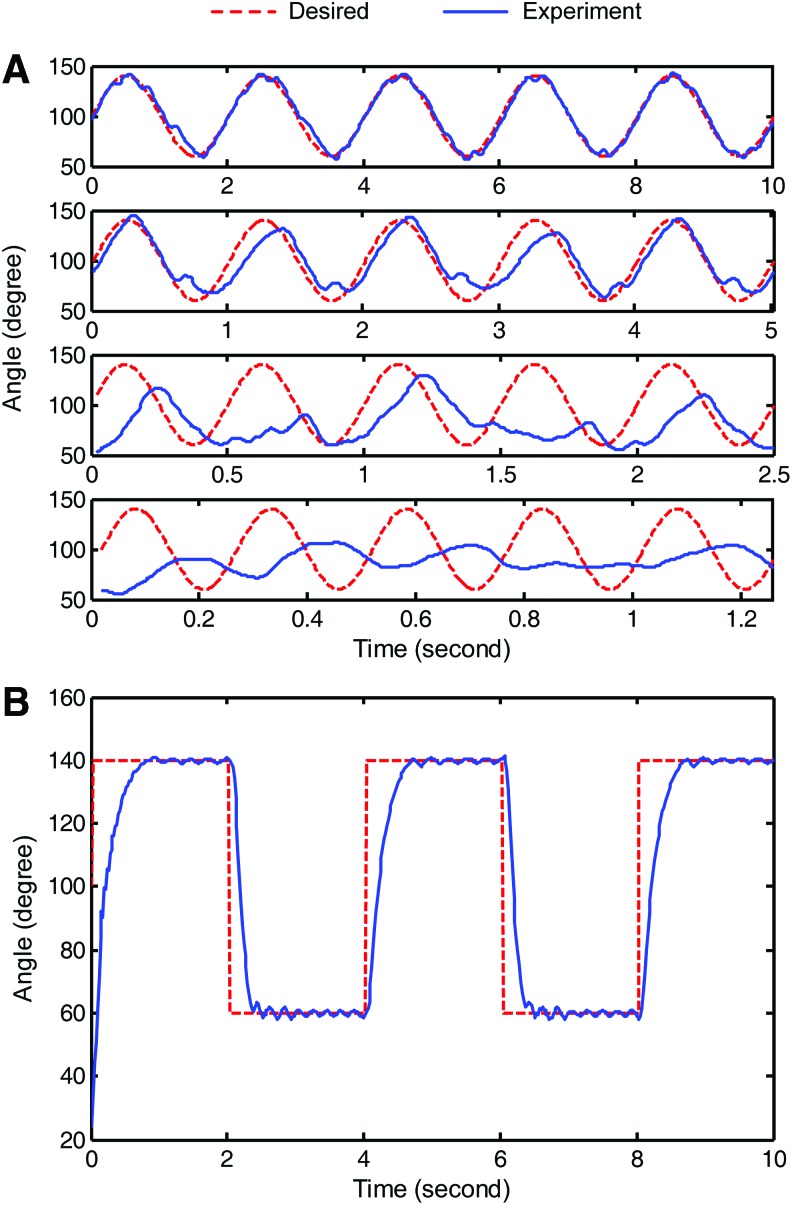
Dynamic performance of the controller in different conditions. **(A)** Sinusoidal wave with a frequency of 0.5, 1, 2, and 4 Hz from *top row* to *bottom row*, respectively; the amplitude is 40° for all frequencies. **(B)** Square wave with a frequency of 0.25 Hz and an amplitude of 40°. Color images are available online.

Second, the sine wave and square wave were used as the desired trajectory to test the controller performances on frequency tracking and amplitude response, respectively. The results on following the 0.5–4 Hz sine waves at an amplitude of 40° are shown in Figure 12A. As shown, the controller performs well in a lower frequency (<1 Hz). When the frequency further increases, the phase delay and amplitude decrease appear great. The phase delay of 0.1 s and amplitude decrease of 20° (∼50%) are observed at a frequency of 4 Hz. Because the overall performance of the used pneumatic valve on bandwidth and air flow can fully support the high-frequency motion, the weak performance of the controller on high frequency mainly comes from the overdamping characteristic of the system model. The material owned stress, which damps the configuration restoring speed, also plays a significant role. As for the local fluctuation observed in tracking the wave with a frequency of 1 Hz, the system model error and the switch of sliding surface in the controller are mainly responsible for their emergence.

Figure 12B shows the controller following the 4 s period square wave at an amplitude of 40°. This frequency is lower than the frequencies used in the previous sine wave tracking tests, which allow the soft actuator to reach the desired angle and hold. We can observe from the result that the step response from a lower angle to the upper angle with amplitude 80° occurs in ∼0.6 s and returns back in ∼0.3 s. When reaching the desired angle, the controller is capable of holding constant angles indefinitely without significant error. No obvious overshoot and oscillation are found along the time.

## Conclusion

In this article, we have presented the design and fabrication of a bidirectional bending pneumatic soft actuator with embedded curvature sensor (optical waveguide made of PMMA material). The main design parameters of the sinusoidal wave chamber, that is, pitch and the amplitude, which significantly affect the bending ability of the soft actuator, are analyzed by FEA method. The simulation result shows that the greater amplitude and smaller pitch endow the soft actuator better bending ability. With proper selection of actuator parameters, the bending angle of the soft actuator can reach 280° under only 0.1 MPa pressure. This shows the potential ability of the proposed actuator in obtaining impressive bending performance. What's more, the output force experiment also indicates that the prototype actuator can obtain favorable moment in relatively low pressure.

The plastic optical waveguide for sensing the curvature shows the superiority of robust sensing of curvature under different pressure. It overcomes the weakness of sensors made of elastomer or conductive liquid that suffers from the crimped surface deformation that likely introduces the unexpected output nonlinearity. The lateral scanning experiments validate the sensitivity of the plastic optical waveguide on external disturbance. The superiorities of steady and linear output voltage corresponding to the increased curvature provide reliable information for closed-loop feedback control.

The dynamic model of the soft actuator is validated by the comparison of the model output against the experimental data under step input in different pressure. Although reducing the system order introduces some fitting error, the model can effectively reproduce the actuator's response to pressure input with acceptable accuracy. The Taylor expansion is used to locally linearize the control variable in designing the sliding mode controller, which requires the control variable to be tuned in small range around the initial value. However, the soft actuator can still be well controllable to track sine wave with an amplitude of 40° and frequency of 1 Hz.

The proposed bidirectional bending soft actuator with curvature proprioceptive ability can be used in a variety of gripper-related applications in industry and medical fields, such as the universal gripper for automatic fruit sorting in food production, exoskeletal glove for hand rehabilitation of stroke survivor, and prosthetic hand for an amputee. In medical field, the bidirectional bending capacity is favorable to cover multisymptoms from early to middle stage of a stroke survivor, such as flaccid paralysis patient whose hand cannot actively flex and dystonia patient whose hand cannot actively open. The prosthetic hand with variable stiffness fingers is another potential application of the proposed soft actuator. The finger's angle and force sensitivity to the external environment are favorable for an amputee. Furthermore, the good performance of the controller is helpful in precise grasping and manipulation for using soft prosthetic hand in daily living.

Toward these potential applications in industry and medical field, future work will focus on force proprioceptive ability, more robust controllers based on system model without precise information, and adaptive control of force and stiffness.
